# Anti-inflammatory and antioxidant properties of oleuropein in human keratinocytes characterized by bottom-up proteomics

**DOI:** 10.3389/fphar.2024.1496078

**Published:** 2025-01-08

**Authors:** Huifang Li, Ni Deng, Jiayi Yang, Yang Zhao, Xiaoxuan Jin, Ang Cai, Navindra P. Seeram, Hang Ma, Dongli Li, Huilan Yang, Chang Liu

**Affiliations:** ^1^ Bioactive Botanical Research Laboratory, Department of Biomedical and Pharmaceutical Sciences, College of Pharmacy, University of Rhode Island, Kingston, RI, United States; ^2^ Proteomics Facility, College of Pharmacy, University of Rhode Island, Kingston, RI, United States; ^3^ Outpatient Department, Southern Theater Command General Hospital, Guangzhou, China; ^4^ School of Pharmacy and Food Engineering, Guangdong Provincial Key Laboratory of Large Animal Models for Biomedicine, Wuyi University, Jiangmen, China

**Keywords:** oleuropein, skin, antioxidant, inflammasome, caspase, proteomics

## Abstract

Oleuropein is a phenolic compound commonly found in cosmetic ingredients including olive leaves and jasmine flowers with various skin-beneficial effects. Here, we evaluated oleuropein’s anti-inflammatory and antioxidant activities in human skin cells. In a cell-based inflammasome model with human monocytes (THP-1 cells), oleuropein (12–200 µM) reduced proinflammatory cytokine interleukin (IL)-6 by 38.8%–45.5%, respectively. Oleuropein (50 and 100 µM) also alleviated oxidative stress in keratinocytes (HaCaT cells) by reducing H_2_O_2_-induced cell death by 6.4% and 9.2%, respectively. Additionally, biological evaluations revealed that oleuropein’s antioxidant effects were attributed to its mitigation of reactive oxygen species in HaCaT cells. Furthermore, a multiplexed gene assay identified IL-1β and thioredoxin-interacting proteins as potential molecular targets involved in oleuropein’s protective effects in HaCaT cells. This was supported by findings from several cellular assays showing that oleuropein reduced the level of IL-1β and inhibited the activity of caspase-1/IL-1 converting enzyme, as well as ameliorated pyroptosis in HaCaT cells. Moreover, a bottom-up proteomics study was conducted to explore potential molecular targets and signaling pathways involved in oleuropein’s antioxidant activities. Taken together, findings from this study expand the understanding of oleuropein’s skin protective effects against oxidative and inflammatory stresses, which support that oleuropein is a promising natural cosmeceutical for skincare applications.

## 1 Introduction

Keratinocytes are the primary cells of the epidermis, the outermost layer of human skin, responsible for maintaining the immune barrier and promoting healing in response to both external (e.g., open wounds) and internal (e.g., free radicals and toxins) stressors (Piipponen et al., 2020). Compromised keratinocytes can lead to impaired skin barrier function and exacerbate signs of aging, such as fine lines and wrinkles. Consequently, there has been significant research interest in identifying compounds that protect keratinocytes from oxidative and inflammatory stress. Natural products derived from medicinal plants and functional foods have emerged as promising bioactives for skin protection. Among these, oleuropein (OLE), a phenolic compound found in olives and jasmine, has demonstrated various skin benefits. For example, OLE has been shown to enhance wound healing by increasing vascular endothelial growth factor (VEGF) and promoting angiogenesis, thus facilitating the formation of new blood vessels at wound sites (Allaw et al., 2021). Additionally, OLE reduces cell infiltration to wound sites, increases collagen fiber deposition, and accelerates the replacement of lost or damaged epithelial cells (Omar, 2010). The wound-healing properties of OLE are primarily attributed to its antioxidant activity, which reduces free radicals in skin tissues, aiding in wound recovery. OLE also exhibits anti-inflammatory effects, as demonstrated in a diabetic mouse model (Liu et al., 2022). Although our laboratory previously reported that OLE confers cytoprotective effects in human dermal fibroblast cells by mitigating H_2_O_2_-induced oxidative stress (Li et al., 2022), the specific molecular targets (i.e., genes and proteins) involved in OLE’s cytoprotective effects on skin remain unclear. Therefore, the current study aims to characterize the antioxidant and anti-inflammatory effects of OLE in human keratinocytes (HaCaT cells) and to identify potential biomarkers, including genes and proteins, that contribute to its cytoprotective activities. We employed a combination of biochemical methods, such as a multigene plex assay, and a bottom-up proteomic approach to identify genes and proteins that may play critical roles in OLE’s biological activities, providing a comprehensive analysis of its mechanisms of action.

## 2 Materials and methods

### 2.1 Chemicals

Oleuropein (OLE) was purchased from Cayman Chemical (Ann Arbor, MI, United States). 2′,7′-dichlorofluorescin diacetate (DCFDA), dimethyl sulfoxide (DMSO), phorbol 12-myristate 13-acetate (PMA), and hydrogen peroxide (H_2_O_2_) solution were purchased from Sigma Aldrich (St. Louis, MO, United States). Cell Titer-Glo^®^ (CTG) 2.0 assay kit was purchased from Promega (Fitchburg, WI, United States). CyQUANT™ XTT Cell Viability Assay kit from Thermo Fisher Scientific (Waltham, MA, United States).

### 2.2 Cell culture and viability

Human monocyte THP-1 cells and Human keratinocyte HaCaT cells were purchased from the American Type Culture Collection (Rockville, MD, United States) and grown in Roswell Park Memorial Institute (RPMI) 1,640 medium and Dulbecco’s modified Eagle’s medium (DMEM) (Life Technologies, Gaithersburg, MD, United States) with 10% fetal bovine serum (FBS) (Life Technologies) at 37°C and 5% CO_2_ at constant humidity. OLE was dissolved in DMSO (dimethyl sulfoxide) and then diluted with a cell culture medium to the desired concentrations. The cytotoxicity of OLE in THP-1 monocytes was measured using an XTT assay. Briefly, THP-1 monocytes were seeded into a 96-well plate at 1×10^4^ cells per well and incubated with PMA (25 nM) for 48 h, then changed to PMA-free medium for another 24 h. Then cells were treated with OLE (at concentrations of 25, 50, 100, and 200 µM) in the presence or absence of LPS (100 ng/mL) for 24 h. The XTT regent (50 μL) was added into each well and incubated for 4 h then the absorbance of each well was recorded at the wavelengths of 492 and 650 nm using a SpectraMax M2 plate reader (Molecular Devices, Sunnyvale, CA). The cytotoxicity of OLE in a model of H_2_O_2_-induced cytotoxicity in HaCaT cells was measured using a CTG 2.0 assay. HaCaT cells were seeded in 96-well plates at 6×10^3^ cells per well and allowed to attach, then incubated with OLE for 2 h and incubated for 22 h without or with H_2_O_2_ (200 µM) (Liu et al., 2019). Next, CTG 2.0 reagent (100 µL) was added to each well and shaken at 200 rpm for 2 min on an orbital shaker. The plate was then incubated at 37°C for 20 min, followed by measuring the luminescence intensity of each well using a plate reader.

### 2.3 Measurement of cytokines

The anti-inflammasome activity of OLE was evaluated by measuring the LPS- and nigericin-induced cytokines according to a reported method (Liu et al., 2020). Briefly, THP-1 cells were seeded at a density of 5 × 10^4^ cells per well in a 48-well plate with PMA (25 ng/mL) for 48 h, then incubated with PMA-free complete medium for 24 h. Next, OLE (at concentrations of 25, 50, 100, and 200 μM) was incubated with cells for 1 h followed by adding LPS (100 ng/mL; incubation for 4 h) then added nigericin (10 μM) for 1 h. Cell culture supernatant was collected to measure IL-1β and IL-6 by the ELISA kits (BioLegend, San Diego, CA, United States).

### 2.4 Non-contact co-culture model with HaCaT and THP-1 cells

HaCaT and THP-1 cells were co-cultured as previously reported (Ma et al., 2016). Briefly, THP-1 monocytes were seeded into a 48-well plate at 2.5 × 10^4^ cells per well and incubated with PMA (25 nM) for 48 h, then changed to PMA-free medium for another 24 h. Then cells were treated with OLE (at concentrations of 25, 50, 100, and 200 µM) for 1 h in the presence or absence of LPS (100 ng/mL) for 1 h, then added nigericin (10 µM) for 1 h and got the supernatant. HaCaT cells were plated in 96-well plates at a density of 6 × 10^3^ cells/mL and incubated it overnight, then removed and replaced with the supernatant from the THP-1 cells for 24-h incubation. The cell viability of HaCaT cells was evaluated by an XTT assay.

### 2.5 Measurement of reactive oxygen species (ROS)

HaCaT cells were seeded in 96-well plates at 6 × 10^3^ cells per well and allowed to attach, then incubated with test samples (at 12.5–100 µM) for 24 h. Next, the cell culture medium was removed and 100 µL medium containing a fluorescent probe 2′,7′-dichlorofluorescin diacetate (DCFDA; 20 μM) was added to the cells and incubated for 20 min. Then cells were treated with 100 µL H_2_O_2_ (200 µM) for 1 h, followed by measuring the fluorescence intensity of each well with excitation and emission wavelengths of 485 and 525 nm, respectively, by a plate reader. Flow cytometry-based assays with specific staining reagents were used to detect ROS levels from mitochondria as we previously reported (Liu et al., 2021). Briefly, HaCaT cells were seeded in 6-well plates at 5 × 10^5^ cells per well and allowed to attach, then the medium was removed, and cells were incubated with OLE (100 μM) for 6 h. Next, cells were stimulated with H_2_O_2_ (200 μM) for 1 h and then stained with MitoSOX reagent for 30 min. The fluorescent intensity of cell suspensions was quantified using flow cytometry (BD FACS Calibur; San Jose, CA, United States) and data were analyzed using the software FlowJo (Ashland, OR, United States).

### 2.6 Quanti-geneplex assay

The multiplexed gene expression assay was performed according to the kit instruction of QuantiGeneTM plex gene expression assay kit (Thermo Fisher Scientific; Waltham, MA, United States). The expression of transcripts related to inflammation pathways was measured using a customized QuantiGene panel in HaCaT cells. The cells were seeded in 48-well plates at 5 × 10^4^ cells per well and allowed to attach. Then the medium was removed, and cells were incubated with OLE (100 μM) for 6 h. Next, a working lysis mixture was added and incubated at 55°C for 30 min, and then cell lysis was hybridized with specific target probe sets for 12 h. The signal of each gene was then generated and amplified by the SP (*Streptavidin phycoerythrin*) fluorescence amplification assay. The mean fluorescence intensity was quantified using a Bio-plex 200 instrument (BioRad; Hercules, CA, United States).

### 2.7 Measurement of cellular IL-1β

HaCaT cells were seeded in 6-well plates at 5 × 10^5^ cells per well and allowed to attach for 12 h. Cells were then incubated with OLE (12.5–100 μM) for 6 h and stimulated with H_2_O_2_ (200 μM) for 24 h, and the cells were collected to extract cell proteins. The level of cellular IL-1β was determined using an ELISA kit (Biolegend; San Diego, CA, United States) according to the manufacturer’s instructions.

### 2.8 Caspase-1 enzyme activity assay

A caspase-1/ICE colorimetric assay kit (R&D Systems; Minneapolis, MN, United States) was used to evaluate the activity of the caspase-1 enzyme. HaCaT cells were cultured in 6-well plates at 5 × 10^5^ cells per well for 24 h and then collected in a conical tube. The cell pellet was lysed with cold lysis buffer (25 μL/1 × 10^6^ cells) and incubated at 4°C for 10 min. The enzymatic reaction for caspase activity was performed in a 96-well microplate containing cell lysate (50 μL), reaction buffer (containing 1% dithiothreitol; 50 μL), and caspase-1 enzyme substrate (YVAD-Pna; 5 μL). The reaction was incubated in the presence or absence of OLE (100 μM) at 37°C for 2 h and the optical density of each well was recorded using a plate reader at a wavelength of 405 nm.

### 2.9 Lactate dehydrogenase assay

Pyroptosis was measured by a lactate dehydrogenase (LDH) assay purchased from Sigma Aldrich. Briefly, HaCaT cells were seeded in 96-well plates at 6 × 10^3^ cells per well and incubated for 12 h. Next, the cells were treated with OLE (12.5–100 μM) for 6 h and followed by incubation with H_2_O_2_ (200 μM) for 24 h. The LDH master reaction mix (50 μL) containing the LDH assay buffer, LDH substrate, and collected cell supernatant was kept on a horizontal shaker in the dark for 2 min. Then the plate was incubated at 37°C for 5 min and the absorbance of each well was recorded at a wavelength of 450 nm using a plate reader.

### 2.10 Sample preparation for bottom-up proteomics analysis

Cell homogenization was performed after cell harvest and protein concentration was determined using a quantification assay. Protein extraction was conducted using a chloroform/methanol extraction method, followed by in-solution trypsin digestion, according to a standardized protocol previously established by our proteomics facility (Puopolo et al., 2024).

### 2.11 Liquid chromatography with tandem mass spectrometry (LC-MS/MS) analysis

Sequential window acquisition of all theoretical mass spectra (SWATH-MS) was performed on an AB Sciex TripleTOF 5,600 mass spectrometer with a DuoSpray™ ion source, coupled to an Acquity H Class HPLC system. An Acquity UPLC Peptide BEH C18 column (2.1 × 150 mm, 300 Å, 1.7 μm) with a VanGuard pre-column (2.1 × 5 mm, 300 Å, 1.7 μm) was used for sample separation at 50°C. The autosampler was maintained at 10°C. Separation was achieved using a 180-min gradient at a flow rate of 10 μL/min, with mobile phases consisting of 0.1% formic acid in water (A) and 0.1% formic acid in acetonitrile (B). The gradient was as follows: 98% A (0–5 min), 98%–70% A (5–155 min), 70%–50% A (155–160 min), 50%–5% A (160–170 min), and 5%–98% A (170–175 min), followed by column re-equilibration. β-galactosidase was injected every four samples for TOF 5600 mass calibration. This method has been extensively used as reported in our previous publications (Puopolo et al., 2023).

### 2.12 Direct data-independent acquisition (DIA) analysis

DirectDIA analysis was conducted using spectral libraries generated from the *Homo sapiens* (Human) FASTA file from UniProt (A9X5H6). Digestion was performed with Trypsin/P, allowing up to two missed cleavages, and peptide lengths were set between 7 and 52 amino acids. Tolerance parameters were set to 1 for both MS1 and MS2 for calibration and the main search. The precursor Q-value cutoff was 0.01, and the precursor PEP cutoff was 0.2. Variable modifications included N-terminal acetylation and methionine oxidation. The False Discovery Rate (FDR) was controlled at 0.01 at the peptide, protein, and peptide-spectrum match (PSM) levels. MS2 signal intensities of fragment ions were obtained from DIA data using Spectronaut™ Pulsar (version 18.0, Biognosys AG) with default settings. The report, exported in a. tsc format, was then used as input for the msDiaLogue package (https://github.com/uconn-scs/msDiaLogue). In the msDiaLogue package, a stringent filtering step was applied to exclude proteins identified by fewer than two peptides (stripped sequences), ensuring the reliability of protein identifications. Peptide (Stripped Sequence): This term refers to the amino acid sequence of a peptide, independent of its charge state or post-translational modifications. It is primarily used for protein inference in mass spectrometry-based proteomics. Additionally, an unpaired t-test was employed to compare two groups, and volcano plots were generated using a fold-change cut-off of 1.5 (log2 = 0.58) and a *p*-value threshold of <0.05.

### 2.13 Bioinformatic analysis

Ingenuity Pathway Analysis (IPA, Qiagen, version 01-2022-01) was performed to assess downstream event changes. Fold changes for the comparisons of Model vs Control and OLE treatment vs Model, along with corresponding *p*-values, were input into IPA to build the database. Fisher’s Exact Test was used to identify associations between the input data and the established annotations within the IPA database, generating insights into canonical pathways, disease functions, and upstream regulators relevant to the dataset. The directionality of expression changes in the dataset was compared with expected patterns from literature annotations, and z-scores were calculated to determine the statistical significance of these predictions.

### 2.14 Statistical analysis

Data were shown as mean ± standard deviation (S.D) from three replicates of experiments. Statistical analysis was performed using GraphPad Prism 10 (GraphPad Software; La Jolla, CA, United States) using one-way analysis of variance with multiple comparisons. A *p*-value of less than 0.05 was considered as a statistical significance between the two groups.

## 3 Results and discussion

### 3.1 OLE inhibits LPS-nigericin-induced IL-6 secretion in THP-1 monocytes

**FIGURE 1:**
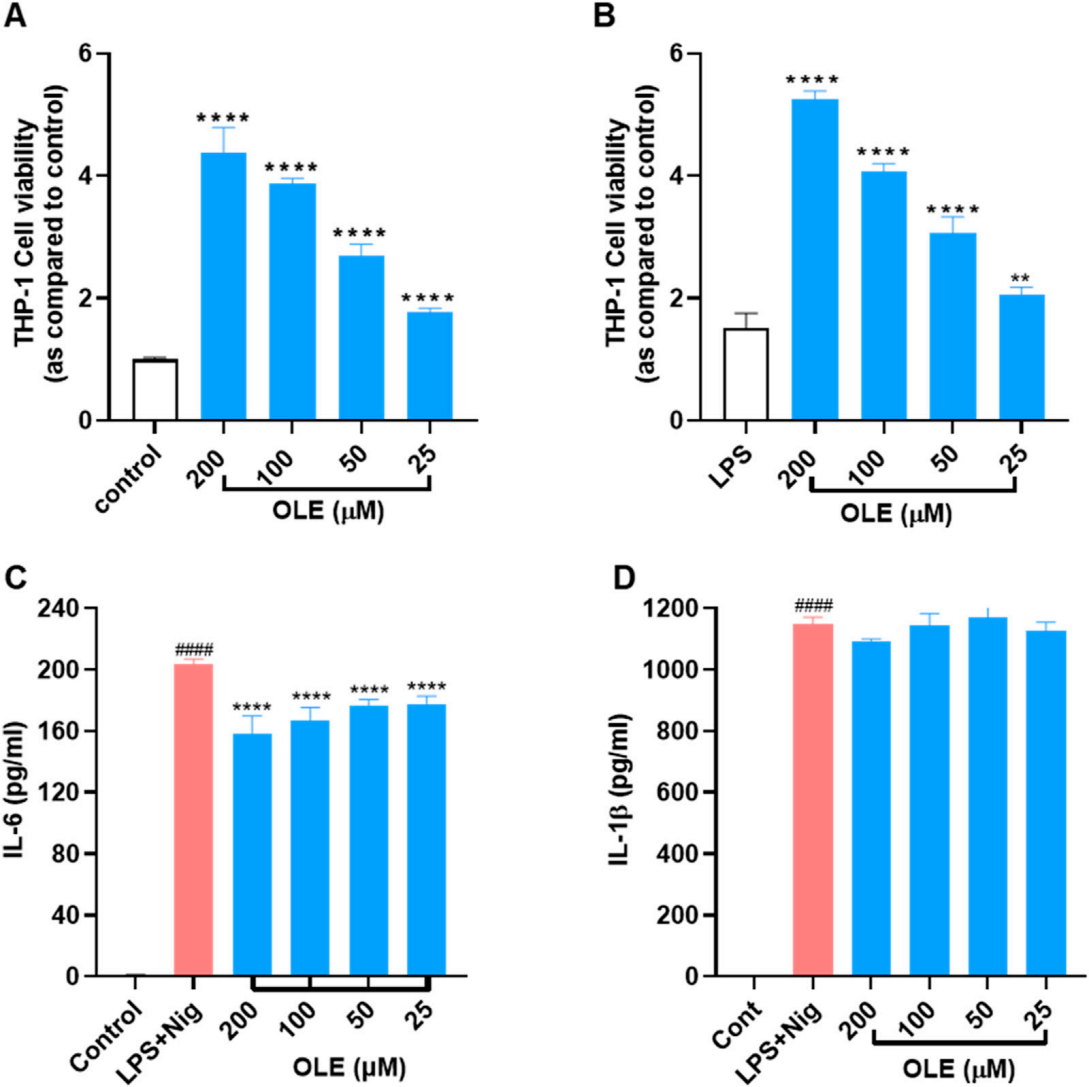
Effects of OLE on the viability of THP-1 cells **(A)** without or **(B)** with the presence of LPS. **p < 0.01, ****p < 0.0001 compared with or without the LPS-treated group. Effects of OLE on the LPS and nigericin-induced secretion of cytokines IL-6 **(C)**, and IL-1β **(D)** in THP-1 monocytes. ^####^ *p* < 0.0001 as compared with control group, ****p < 0.0001 as compared with LPS + Nig group.

We first evaluated OLE’s effects on the viability of THP-1 monocytes with and without the presence of LPS. Treatment with OLE (25, 50, 100, and 200 μM) was not toxic to THP-1 monocytes as the cell viability was higher than the control group’s (Figure 1A). Similarly, in the presence of LPS (100 ng/mL), there were no signs of cytotoxicity with the treatment of OLE (25–200 μM, cell viability >100%) (Figure 1B). Next, the anti-inflammasome activity of OLE in THP-1 monocytes was evaluated in an inflammasome model induced by LPS-nigericin. Stimulation with LPS-nigericin significantly increased the concentration of IL-1β and IL-6 in THP-1 monocytes from 1.5 to 1,146.6 pg/mL and from 0.7 to 203.5 pg/mL, respectively, compared to the control group. Treatment with OLE (25, 50, 100, and 200 μM) decreased the IL-6 secretion to 158.4, 167.3, 176.8, and 177.8 pg/mL, respectively (Figure 1C), while not significantly lowering the expression level of IL-1β (Figure 1D).

### 3.2 OLE protects HaCaT cells from inflammatory and oxidative stress

**FIGURE 2:**
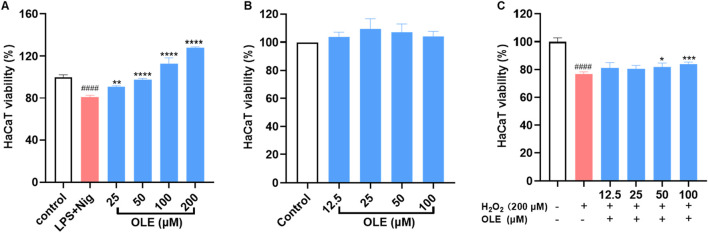
Protective effects of OLE on LPS and nigericin-THP-1 conditioned media induced cytotoxicity of HaCaT cells **(A)**. Effects of OLE on the viability of HaCaT cells without **(B)** or with **(C)** H_2_O_2_. ^####^ *p* < 0.0001 compared with the control group; *p < 0.05, **p < 0.01, ***p < 0.001, ****p < 0.0001 as compared with the H_2_O_2_-treated or LPS + Nig group.

Co-culture models have been employed to investigate the impact of THP-1 cells and their inflammatory mediators on the cell death of HaCaT cells. Thus, we used a non-contact cell culture model with conditioned media to evaluate the protective effects of OLE against LPS-nigericin-induced inflammation and cytotoxicity. OLE reduced the cytotoxicity of HaCaT cells exposed to a conditioned medium from THP-1 cells. HaCaT cells exposed to THP-1 cell media showed reduced viability by 18.9% compared to the control group, while OLE at concentrations of 25, 50, 100, and 200 µM restored the HaCaT cell viability by 12.1%, 20.5%, 39.3%, and 59.0% compared to the model group, respectively (Figure 2A). Next, the cytoprotective effects of OLE against H_2_O_2_-induced oxidative stress in HaCaT cells were evaluated. In the model group, cells exposed to H_2_O_2_ (200 μM) had reduced cell viability by 76.9%. OLE (at concentrations of 12.5, 25, 50, and 100 μM) was non-toxic to HaCaT cells (with >100% cell viability; Figure 2B) and it reduced H_2_O_2_-induced cytotoxicity by increasing the cell viability to 81.3%, 80.5%, 81.8%, and 84.0% at the concentrations of 12.5, 25, 50, and 100 μM, respectively (Figure 2C).

**FIGURE 3:**
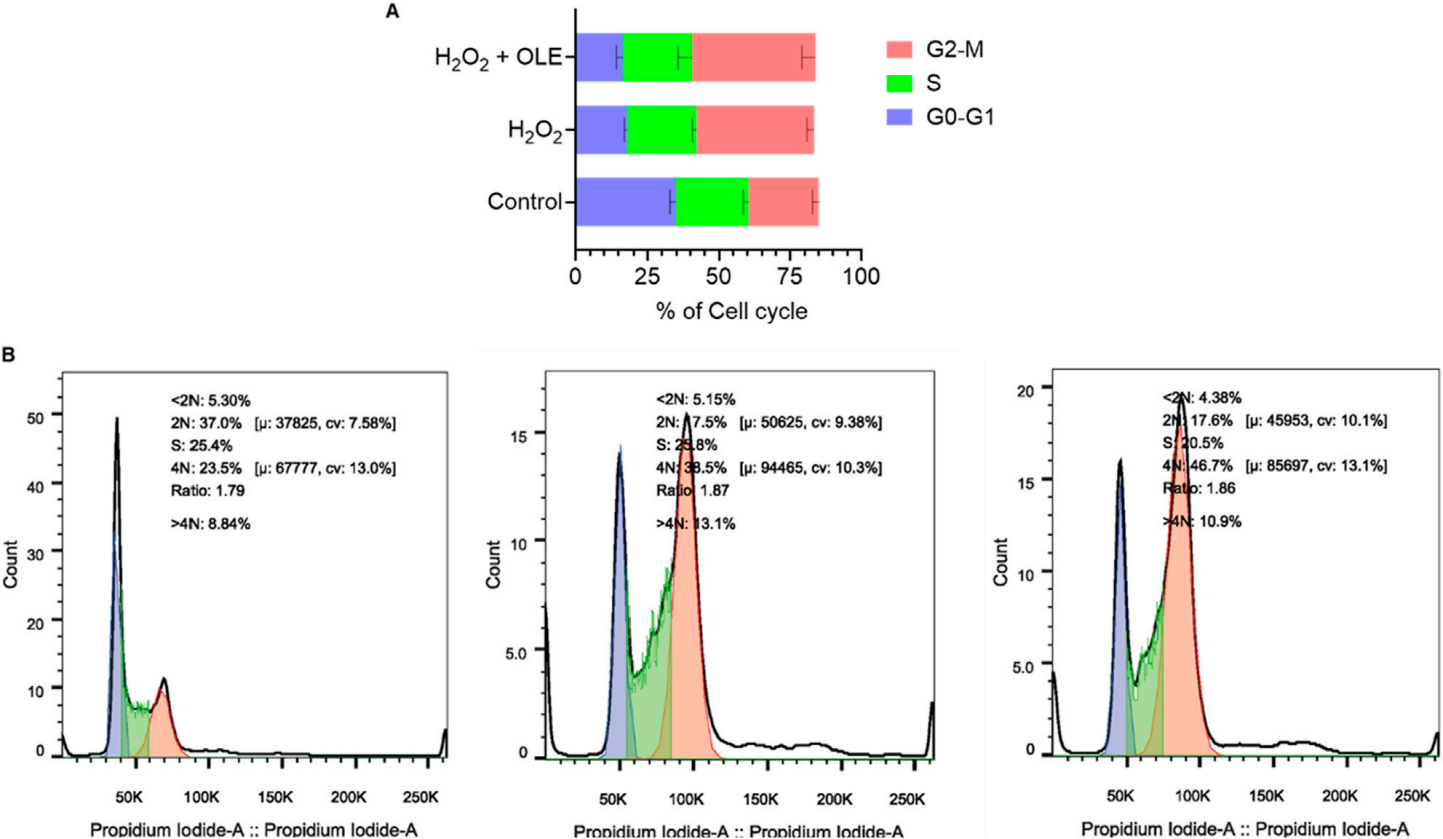
Effect of OLE on the cell-cycle progression in HaCaT cells analyzed by flow cytometry **(A)**, and quantification of the mean fluorescence intensity **(B)**.

This cytoprotective effect was further investigated by assessing the effects of OLE on the cyclic progression of HaCaT cells exposed to H_2_O_2_. The flow cytometric analysis revealed that stimulation with H_2_O_2_ (200 μM) altered cell cycle distribution including phases of G2-M, S, and G0-G1, compared to the control group. Treatment with OLE (100 μM) attenuated H_2_O_2_-induced cell cycle transition as it reduced the S phase by 7.5% (Figure 3).

### 3.3 OLE attenuates H_2_O_2_-induced reactive oxygen species (ROS) in HaCaT cells

**FIGURE 4:**
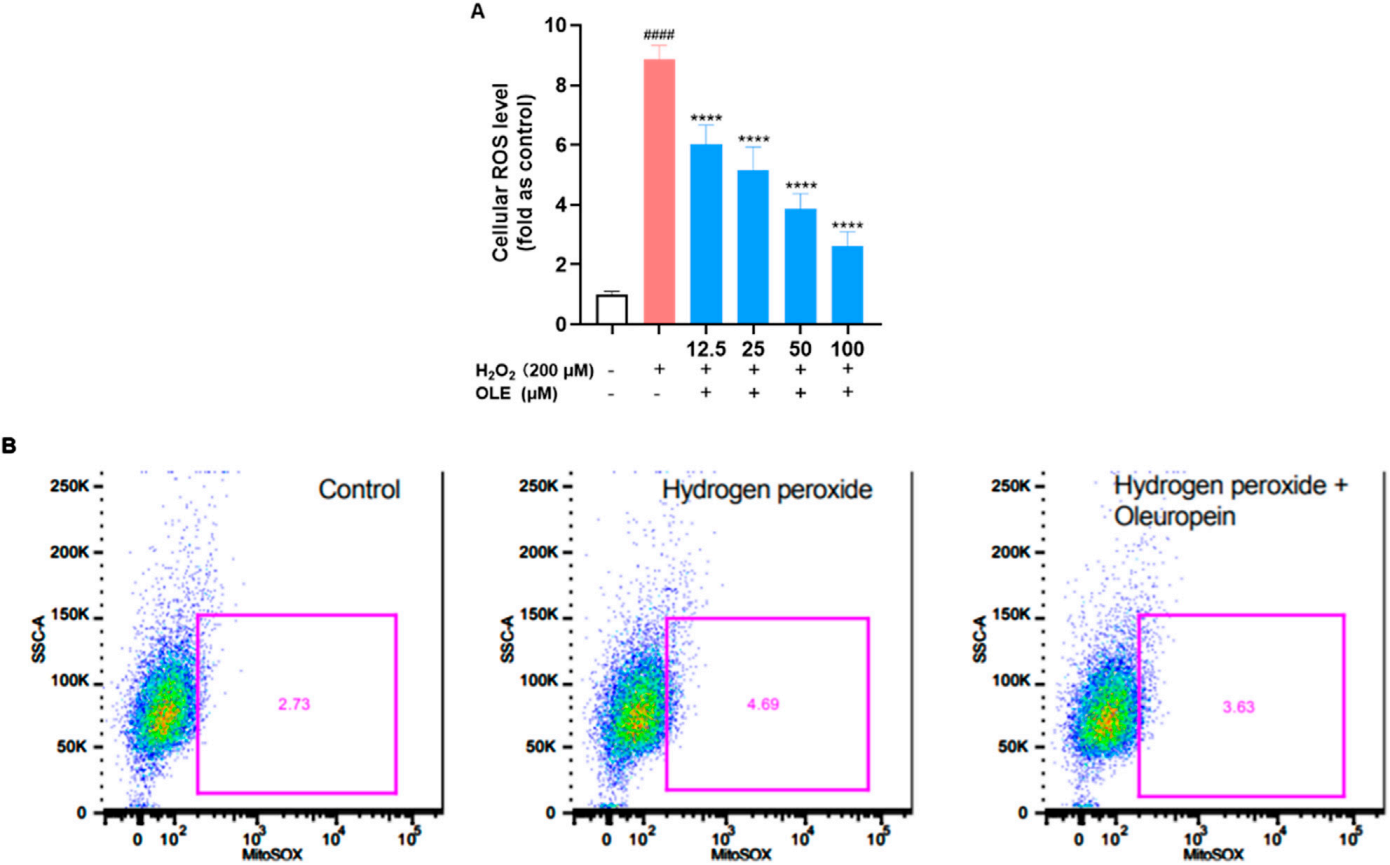
Effects of OLE on the total production of ROS **(A)** and cellular mitochondrial ROS **(B)** in H_2_O_2_-exposed HaCaT cells. ^####^ *p* < 0.0001 as compared with control group, ****p < 0.0001 as compared with H_2_O_2_ group.

To further evaluate the cytoprotective effect of OLE on HaCaT cells, we studied if OLE could attenuate H_2_O_2_-induced ROS in HaCaT cells. A fluorescent probe 2′,7′-dichlorodihydrofluorescein diacetate (DCF-DA) was used to measure the total ROS level in HaCaT. In HaCaT cells, H_2_O_2_-induced oxidative stress increased the production of cellular ROS production by 7.9-fold compared to the control group, while OLE (at concentrations of 12.5, 25, 50, and 100 μM) decreased the formation of the oxidized fluorescent product 2′,7′-dichlorofluorescein to 2.6-, 3.9-, 5.2-, and 6.0-fold, respectively (Figure 4A). Given that mitochondria are responsible for major cellular ROS (Tirichen et al., 2021), we used CellROX, a cell-permeant fluorogenic agent that specifically stains mitochondrial ROS, to assess OLE’s antioxidant effects. As shown in Figure 4B, H_2_O_2_-induced mitochondrial ROS was increased by 135.6% compared to the control group and OLE decreased the mitochondrial ROS by 22.6% compared to the H_2_O_2_ group.

### 3.4 OLE modulates the gene expression of IL-1β and TXNIP

**FIGURE 5:**
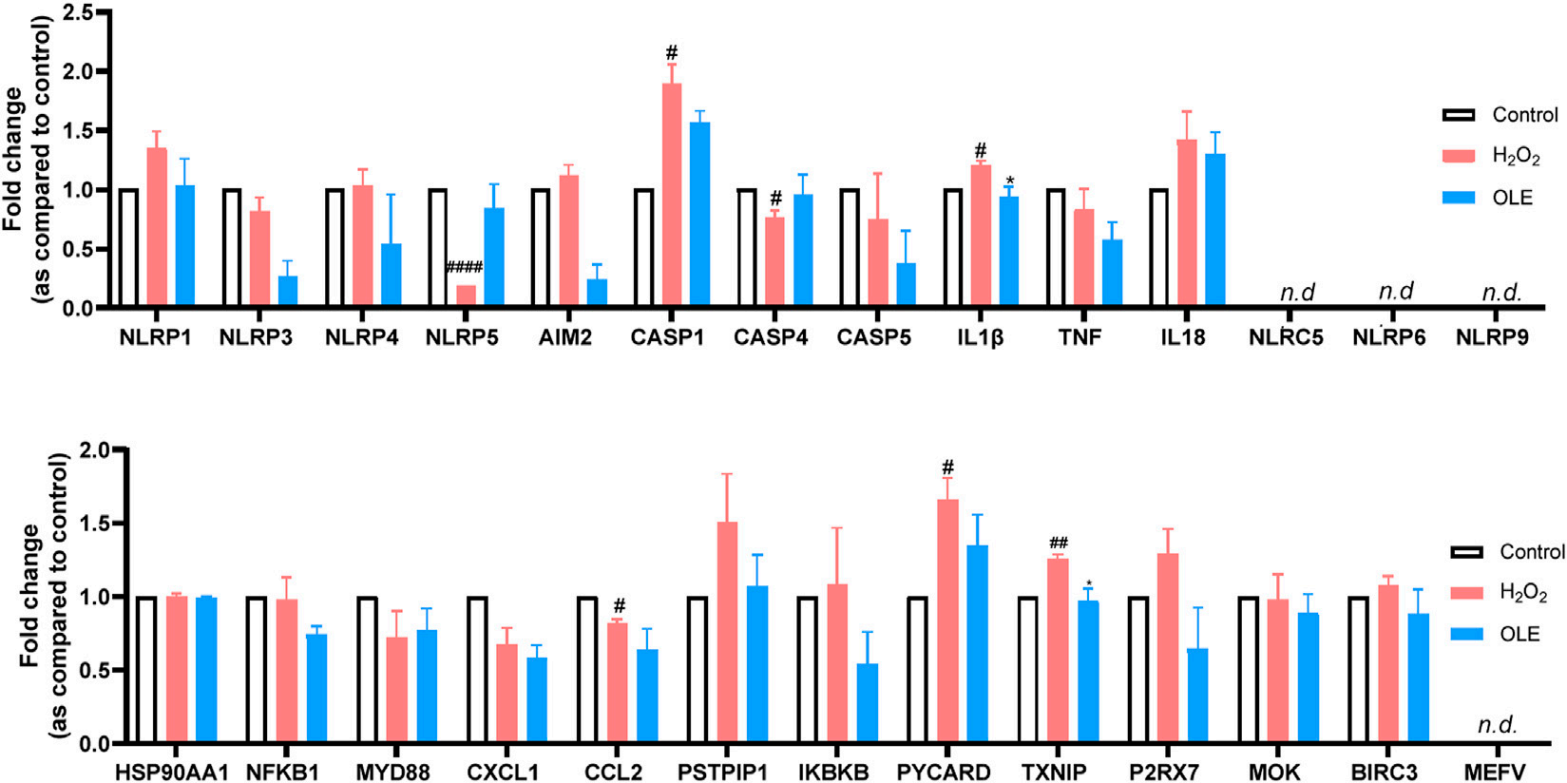
Effects of OLE on the mRNA expression levels of inflammasome-related genes measured by the multiplexed gene expression assay. Statistical analysis used a two-way analysis of variance with multiple comparisons. ^#^ *p* < 0.05, ^##^ *p* < 0.01, ^####^ *p* < 0.0001 as compared with the control group; *p < 0.05 as compared with the H_2_O_2_ group.

Given that excessive cellular ROS may trigger the activation of NLRP3 and lead to the formation of inflammasome complex (Choi and Park, 2023), OLE’s effect on the expression of inflammasome-related genes in HaCaT cells was explored with a multiplexed gene expression assay (see the list of full genes in this panel in the Supplementary Materials). The results revealed that stimulation with H_2_O_2_ increased the mRNA expression levels of genes including IL-1β and TXNIP (thioredoxin-interacting protein) by 19.9% and 25.6%, respectively, compared to the control group (Figure 5). Treatment with OLE downregulated the mRNA expression levels of genes IL1β and TXNIP by 21.6% and 22.8%, respectively, compared to the H_2_O_2_-stimulated cells.

IL-1β is a well-studied pro-inflammatory cytokine that plays a critical role in mediating and amplifying inflammation. It is part of the body’s innate immune response and is produced by a variety of cells, primarily macrophages and monocytes, in response to infections, tissue damage, or other stressors (Lopez-Castejon and Brough, 2011). On the other hand, TXNIP is a key regulator of oxidative stress and cellular redox balance. It can dictate the response to oxidative by several mechanisms including the inhibition of thioredoxin (TRX), activation of NLRP3 inflammasome, and regulation of metabolic pathways (Lane et al., 2013). OLE has been reported to reduce inflammation by modulating NLRP3 inflammasome. For instance, OLE was reported to inhibit murine lupus nephritis NLRP3 inflammasome-related signaling pathways (Castejon et al., 2019). Thus, it is not surprising that OLE treatment significantly reduced the expression of IL-1β in and TXNIP the multiplexed gene assay. However, further functional studies were warranted to confirm OLE’s effects on caspase-1.

### 3.5 OLE decreases cellular IL-1β and caspase-1 level in H_2_O_2_-stimulated HaCaT cells

**FIGURE 6:**
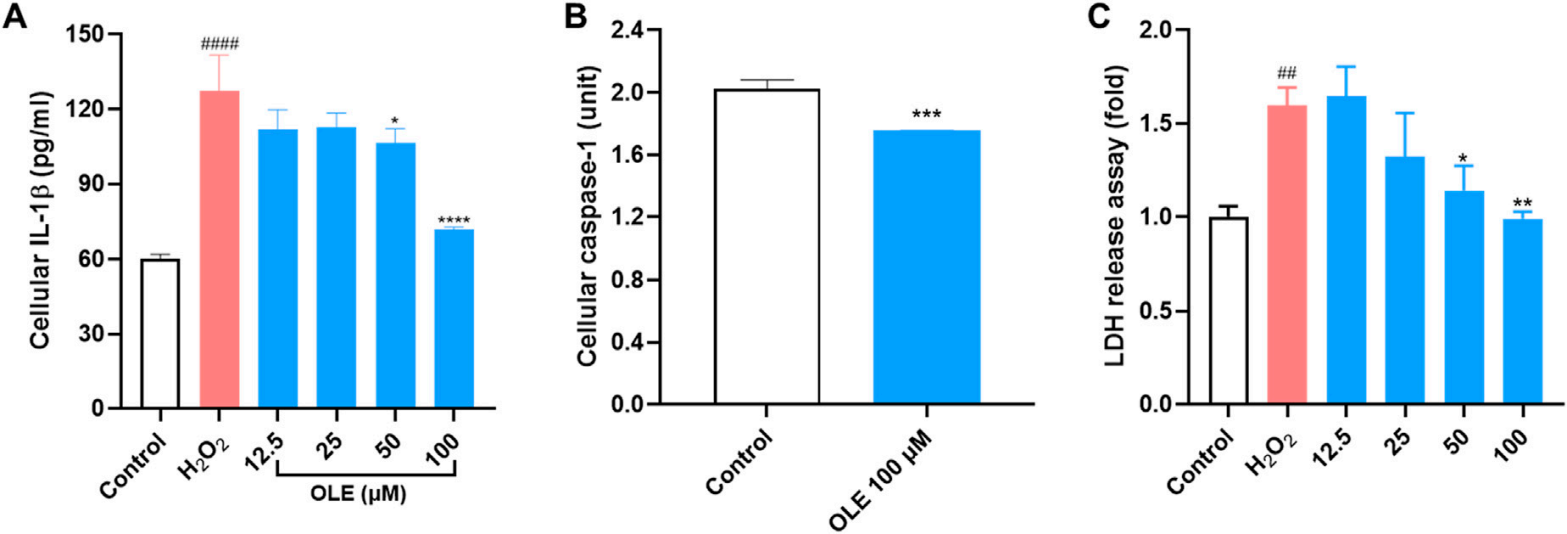
Effects of OLE on the release of cellular IL-1β **(A)**, caspase-1 enzyme activity **(B, C)** LDH release in HaCaT cells. ^####^ *p* < 0.0001 as compared with the control group. *p < 0.05, **p < 0.01, ***p < 0.001, ****p < 0.0001 as compared with the H_2_O_2_-treated group.

Based on the multiplexed gene assay, IL-1β could be a critical gene that regulates OLE’s antioxidant and anti-inflammatory effects in HaCaT cells. Thus, we evaluated OLE’s effects on the cellular IL-1β level in H_2_O_2_-stimulated HaCaT cells. Stimulation with H_2_O_2_ (200 μM) elevated the level of cellular IL-1β in HaCaT cells by 111.3%, which was counteracted by the treatment with OLE (50 and 100 μM) by 11.5%, 12.2%, 16.3%, and 43.8%, respectively (Figure 6A). Additionally, the production of IL-1β is regulated by caspase-1 and it is unknown whether OLE can decrease IL-1β by inhibiting the activity of caspase-1 (a precursor of IL-1β). Therefore, we also evaluated OLE’s inhibitory effect on the activity of caspase-1 enzyme and revealed that OLE inhibited caspase-1 activity by 13.4% (Figure 6B). Next, we evaluated whether OLE alleviated pyroptosis, a form of inflammation-mediated programmed cell death activated by caspase 1 (Man et al., 2017). Several key events including the rapid formation of membrane pores, significant cell swelling, and subsequent membrane rupture, releasing intracellular contents, such as cytosolic proteins (e.g., lactate dehydrogenase; LDH), are associated with pyroptosis (Rayamajhi et al., 2013). Thus, OLE’s effect on H_2_O_2_ (100 μM) elevated LDH production (by 59.8%) in HaCaT cells was assessed. Treatment with OLE (25, 50, and 100 μM) decreased the LDH production by 17.2%, 28.6%, and 38.1%, respectively, compared to the model group (Figure 6C).

### 3.6 OLE induces proteome changes in H_2_O_2_-stimulated HaCaT cells

**FIGURE 7:**
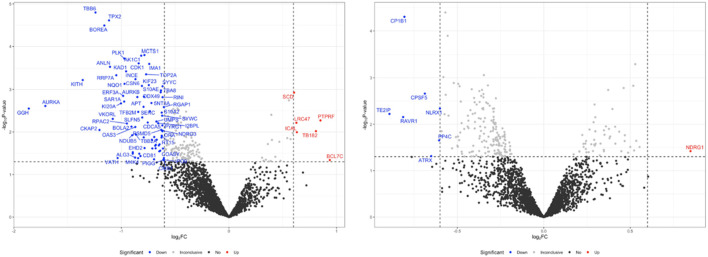
Volcano plot of downregulated and upregulated proteins after H_2_O_2_ stimulation compared to the control group(A), and OLE group compared to the H_2_O_2_ group. The volcano plots were generated using a cut-off value of 0.58 (1.5-fold change) and a *p*-value <0.05. The x-axis represents the log2 fold change, and the y-axis represents the negative log10 *p*-value.

**FIGURE 8:**
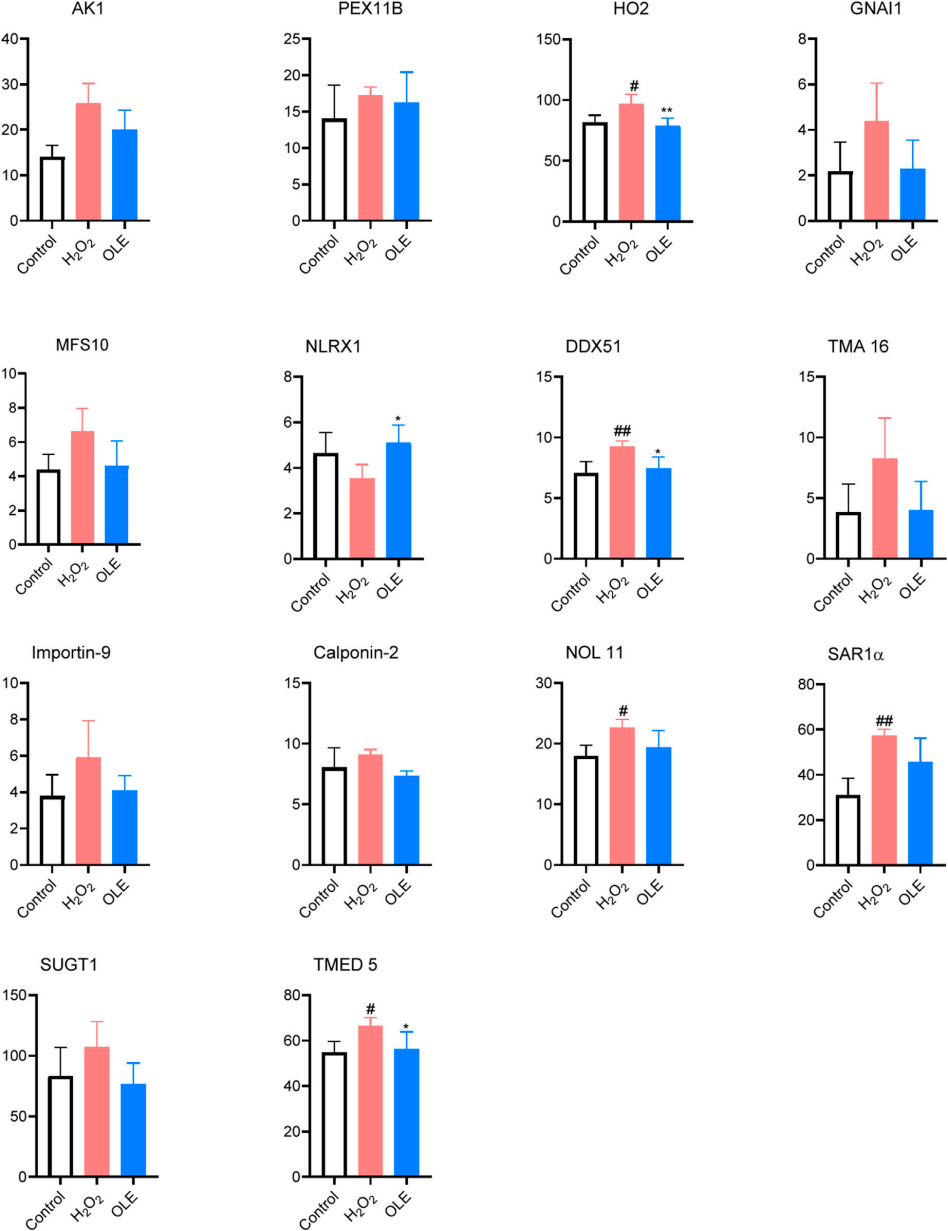
Effects of OLE on protein expression levels of oxidative press-related proteins measured by the proteomics assay. Statistical analysis used a one-way ANOVA analysis of variance with multiple comparisons. ^#^ *p* < 0.05, ^##^ *p* < 0.01 as compared with the control group; *p < 0.05, ^**^ *p* < 0.01 as compared with the H_2_O_2_ group.

We conducted a bottom-up proteomics analysis using SWATH-MS to investigate the impact of OLE on proteome changes in H₂O₂-stimulated HaCaT cells. A DirectDIA analysis initially identified 3,288 proteins. After reprocessing the data with msDiaLogue and applying a stringent filtering step to exclude 777 proteins identified by fewer than two unique peptides, 2,511 reliable proteins were retained. As shown in Figure 7, compared to the control group, there were 77 upregulated and 5 downregulated proteins in H_2_O_2_-stimulated HaCaT cells (Model group). Comparing the OLE treatment group to the Model group, 7 proteins were upregulated and 1 was downregulated. However, when applying a more stringent threshold of a 1.5-fold change (Log2FC > 0.58 and Log2FC <−0.58) and a *p*-value of less than 0.05, no proteins from the OLE treatment group counter-regulated the proteome changes induced by oxidative stress. Overall, we identified fourteen proteins, of which thirteen were upregulated in the Model group and downregulated by OLE, while one was downregulated in the Model group and upregulated by OLE (Figure 8).

Expressions of several proteins including HO2, DDX51, NOL11, SAR1α, and TMED5 were significantly elevated by oxidative stress in the model group (Figure 8). Of these proteins, treatment with OLE counteracted the protein expressions of HO2, DDX51, and TMED5. Several proteins modulated by OLE treatment in response to oxidative stress are potential targets contributed to OLE’s antioxidant effects. The HO2 protein, also known as heme oxygenase-2, plays a significant role in the cellular antioxidant defense system. HO2 can degrade heme, which is a component of hemoglobin, into biliverdin, carbon monoxide, and free iron. The breakdown products, such as biliverdin and its reduced form bilirubin, have potent antioxidant properties (Intagliata et al., 2019). In the model group, cells exposed to H_2_O_2_ had higher expression of HO2 protein, suggesting that this protein could play a critical role in mediating oxidative stress in HaCaT cells. This is in agreement with previously reported studies showing that HO-1 exerts antioxidant effects in human keratinocytes, which is associated with the production of inflammatory cytokines (Numata et al., 2009). Moreover, oleuropein is reported to modulate HO1 protein, an isoenzyme form of HO2, to alleviate oxidative stress in liver tissue (Domitrović et al., 2012).

An ingenuity pathway analysis (IPA) was performed to elucidate the signaling pathways affected by H_2_O_2_ stimulation. Compared to the control group, several top canonical pathways including mitotic metaphase and anaphase (*p* = 2.47 × 10⁻^10^), RHO GTPases activate formins (*p* = 7.34 × 10⁻^10^), and mitotic prometaphase (*p* = 1.67 × 10⁻^9^) in the oxidative stress model were involved (Table 1).

**TABLE 1:** Predicted top canonical pathways were derived from IPA analysis for the control and the oxidative stress model groups.

Top canonical pathways	*p*-value	Overlap
Sirtuin Signaling Pathway	1.65E-11	7.3% (21/286)
Mitotic G2-G2/M	1.45E-10	8.5% (17/199)
Mitotic Metaphase and Anaphase	2.47E-10	7.7% (18/235)
RHO GTPases Activate Formins	7.34E-10	10.1% (14/139)
Mitotic Promephase	1.67E-09	7.9% (16/203)

The IPA results predicted the activation of key upstream regulators such as APP (amyloid-beta precursor protein; *p* = 5.46 × 10⁻^9^), MYC (MYC proto-oncogene; *p* = 1.78 × 10⁻^8^), TGFB1 (transforming growth factor beta 1; *p* = 1.12 × 10⁻^13^), FCER1G (Fc fragment of IgE; *p* = 3.97 × 10⁻^12^), ABL1 (tyrosine-protein kinase ABL1; *p* = 6.17 × 10⁻^12^), and PML (promyelocytic leukemia protein; *p* = 1.47 × 10⁻^11^), while CEACAM1 (carcinoembryonic antigen-related cell adhesion molecule 1; *p* = 8.52 × 10⁻^12^) was predicted to be inhibited by H_2_O_2_ stimulation (Table 2).

**TABLE 2:** Predicted top upstream regulators from IPA analysis for the control and the oxidative stress model groups.

Top upstream regulators or causal network	*p*-value	Predicted activation
APP	5.46E-09	Activated
MYC	1.78E-08	Activated
TGFB1	1.12E-13	Activated
FCER1G	3.97E-12	Activated
ABL1	6.17E-12	Activated
CEACAM1	8.52E-12	Activated
PML	1.47E-11	Activated

Our group has used the proteomics approach to study the molecule pathways in human keratinocytes in the condition of oxidative stress (Li et al., 2023). We optimized this method to study OLE’s mechanisms of action. Among these canonical pathways identified by proteomics, the sirtuin pathway consisting of a family of NAD + -dependent deacetylases is a classic master regulator involved in mediating cellular oxidative stress response. This pathway plays a crucial role in cellular homeostasis by influencing DNA repair, mitochondrial function, and apoptosis (Salminen et al., 2013). Under oxidative stress, sirtuins (particularly SIRT1) modulate the activity of key transcription factors like FOXO and PGC-1α, which control antioxidant defenses. Activation of sirtuins promotes cellular survival by reducing ROS production and enhancing mitochondrial biogenesis. This was in agreement with data from our cellular assays and numerous studies showing that H_2_O_2_ can induce oxidative stress in HaCaT cells by modulating the SIRT pathways (Cao et al., 2009; Lee et al., 2016). Additionally, proteomics analysis identified the mitotic G2-G2/M pathway as an important biomarker for H_2_O_2_-induced oxidative stress in HaCaT cells. This is not surprising given that the mitotic G2-G2/M pathway is a checkpoint that ensures cells with DNA damage do not enter mitosis, allowing time for repair (Wang et al., 2017). Moreover, the role of the mitotic G2-G2/M pathway in response to oxidative stress is supported by data from our flow cytometric assays showing that the G2/M phase of HaCaT cells was affected by exposure to H_2_O_2_. To date, this is the first study showing that the mitotic G2-G2/M pathway is a biomarker for oxidative stress in HaCaT cells. This demonstrates that our proteomics study identified new molecular targets involved in OLE’s response to oxidative stress in human keratinocytes.

Next, our proteomics analysis revealed that OLE treatment regulated several top canonical pathways including the processing of capped intron-containing pre-mRNA (*p* = 1.84 × 10⁻^34^), FAT10 signaling pathway (*p* = 2.67 × 10⁻^31^), regulation of apoptosis (*p* = 6.84 × 10⁻^30^), metabolism of polyamines (*p* = 1.25 × 10⁻^28^), and noncanonical NF-κB signaling (*p* = 1.96 × 10⁻^28^) (Table 3). Additionally, several key upstream regulators such as 5-methyltetrahydrofolic acid (*p* = 1.01 × 10⁻^29^), folic acid (*p* = 1.12 × 10⁻^27^), WTAP (WT1 associated protein; *p* = 4.44 × 10⁻^21^), and NFE2L1 (nuclear factor erythroid 2 like 1; *p* = 7.95 × 10⁻^15^) were predicted to be activated by OLE treatment (Table 4).

**TABLE 3:** Predicted top canonical pathways were derived from IPA analysis for the OLE treatment and the model groups.

Top canonical pathways	*p*-value	Overlap
Processing of capped intro-containing Pre-mRNA	1.65E-11	7.3% (21/286)
FAT10 Signaling Pathway	1.45E-10	8.5% (17/199)
Regulation of Apoptosis	2.47E-10	7.7% (18/235)
Metabolism of polyamines	7.34E-10	10.1% (14/139)
Noncanonical NF-KB signaling	1.67E-09	7.9% (16/203)

**TABLE 4:** Predicted top upstream regulators from IPA analysis for OLE treatment and the model groups.

Upstream regulators or causal network	*p*-value	Overlap
5-methyletrahydrofolic acid	1.65E-11	7.3% (21/286)
Folic acid	1.45E-10	8.5% (17/199)
WTAP	2.47E-10	7.7% (18/235)
NFE2L1	7.34E-10	10.1% (14/139)

Among the identified canonical pathways, the processing of capped intron-containing pre-mRNA pathway involves the processing and splicing of pre-mRNA, which includes adding a 5′cap to protect the RNA from degradation and facilitating splicing by removing introns. Under oxidative stress, splicing can be disrupted due to ROS affecting the spliceosome machinery and RNA-binding proteins. This leads to errors in mRNA processing and potentially abnormal protein synthesis. Oxidative stress-induced damage to RNA itself can also impact this pathway, contributing to cellular dysfunction (Ling et al., 2017). Although this is the first study suggesting that the processing of capped intron-containing pre-mRNA pathway is a potential biomarker for OLE’s antioxidant effects in HaCaT cells, further studies of functional assays are warranted to confirm it. Nevertheless, the proteomics study also identified pathways that have been reported for OLE’s antioxidant activities. For instance, proteomics identified the noncanonical NF-κB signaling involved in OLE’s antioxidant effects in HaCaT cells. Unlike the classical NF-κB pathway, which is rapidly activated by pro-inflammatory signals, the noncanonical NF-κB pathway is activated by specific signals like lymphotoxin, playing a slower role in immune regulation. In oxidative stress, this pathway contributes to cellular survival by regulating genes involved in antioxidant responses, immune function, and inflammation. It can be activated by ROS through kinase signaling cascades, enabling cells to adapt and survive oxidative conditions​ (Lingappan, 2018). Oleuropein has been reported to interfere with NF-κB signaling pathways, reducing inflammation and oxidative stress in peritoneal macrophages (Castejón et al., 2020). This is supported by our experiments showing that OLE downregulated pro-inflammatory cytokines and upregulated antioxidant defenses, which protects HaCaT cells from oxidative damage and chronic inflammation. Furthermore, it was noted that NFE2L1, nuclear factor erythroid 2 like 1 (also known as Nrf1), was identified as a molecular target for OLE’s antioxidative effects. NFE2L1 has been studied as a transcription factor that plays a critical role in cellular responses to oxidative stress. It mediates oxidative stress through several mechanisms including regulation of antioxidant gene expression (e.g., superoxide dismutase and glutathione peroxidase), proteasomal homeostasis, and as a complementary role to NFE2L2 (Nrf2) to compensate for loss of Nrf2 function (Liu et al., 2023). Studies have shown that OLE can induce mitochondrial biogenesis and decrease reactive oxygen species generation in cultured avian muscle cells via the modulation of SIRT1 and Nrf1 gene expression (Kikusato et al., 2016). The current study demonstrates that OLE may modulate NFE2L1 to alleviate oxidative stress in HaCaT cells. Our findings provide evidence for OLE’s skin-protective effects using proteomic analysis, identifying key proteins and pathways involved in its antioxidant and anti-inflammatory actions. However, to confirm these findings, functional assays like Western blotting, immunohistochemistry, and qRT-PCR are needed to validate the proteomic results at the protein and gene expression levels. These assays would strengthen the understanding of OLE’s mechanisms of action, which are critical for its potential development as an active ingredient for skin health.

## 4 Conclusion

In summary, we evaluated the anti-inflammatory and antioxidant activities of OLE in human monocytes and keratinocytes with a panel of biochemical assays. OLE exerts anti-inflammatory effects by reducing the level of pro-inflammatory cytokine in human THP-1 cells. Additionally, in a non-contact co-culture model (with condition cell culture medium from THP-1 cells), OLE protects HaCaT cells by ameliorating inflammatory stress-induced cytotoxicity. Moreover, OLE protects HaCaT cells by reducing ROS production and mediating cell cycles. The plausible mechanism(s) for OLE’s anti-inflammatory and antioxidant activities were explored by a multiplexed gene expression assay, which identified enzymes including caspase-1 as the potential targets. Furthermore, a bottom-up proteomics study characterized a series of canonical pathways and upstream regulators that may contribute to OLE’s overall biological effects. Data from the current study provide useful information to support OLE’s skin protective effects, which expands our understanding of OLE’s effects as a promising cosmeceutical.

## Data Availability

The data generated and analyzed in this study have been deposited in the MassIVE repository (https://massive.ucsd.edu/ProteoSAFe/static/massive.jsp) under the project identifier “MassIVE MSV000096730.” The dataset is accessible via the FTP link: ftp://massive.ucsd.edu/v07/MSV000096730/. For further details or inquiries, please contact CL at hichang813@uri.edu.
