# Supplementary material for: Anti-inflammatory and antioxidant properties of oleuropein in human keratinocytes characterized by bottom-up proteomics
Source: Front Pharmacol. 2025 Jan 8;15:1496078. doi: 10.3389/fphar.2024.1496078 (PMC11751055; doi:10.3389/fphar.2024.1496078)
Supplement: Supplementary file 1 [file Table1.docx]

Supplementary Material

**Table 1.** The list of full genes in the inflammasome 27-plex panel.

| **Gene** | **Description** | **Gene Name** |
| --- | --- | --- |
| NLRX1 | NLR family member X1 | CLR11.3, DLNB26, FLJ21478, MGC131937,  MGC21025, NOD26, NOD5, NOD9 |
| NLRP3 | NLR family, pyrin domain containing 3 | AGTAVPRL, AII, AII, AVP, AVP, C1orf7, CIAS1, CLR1.1, FCAS,  FCU, FLJ95925, MWS, NALP3, PYPAF1 |
| NLRP4 | NLR family, pyrin domain containing 4 | CLR19.5, CT58, FLJ32126,  NALP4, PAN2, PYPAF4, RNH2 |
| NLRP5fr | NLR family, pyrin domain containing 5 | CLR19.8, MATER, NALP5, PAN11, PYPAF8 |
| AIM2 | Absent in melanoma 2 | PYHIN4 |
| CASP1 | Caspase 1, apoptosis-related cysteine peptidase (interleukin 1, beta, convertase) | ICE, IL1BC, P45 |
| CASP4 | Caspase 4, apoptosis-related cysteine peptidase | ICE(rel)II, ICEREL-II, ICH-2, Mih1, TX, TX |
| CASP5 | Caspase 5, apoptosis-related cysteine peptidase | ICE(rel)III, ICEREL-III, ICH-3, MGC141966 |
| IL1 | Interleukin 1, beta | IL-1, IL1-BETA, IL1F2 |
| TNF | Tumor necrosis factor (TNF superfamily, member 2) | DIF, TNF-alpha, TNFA, TNFSF2 |
| IL18 | Interleukin 18 (interferon-gamma-inducing factor) | IGIF, IL-18, IL-1g, IL1F4, MGC12320 |
| NLRC5 | NLR family, CARD domain containing 5 | CLR16.1, FLJ21709, FLJ39711, NOD27, NOD4 |
| NLRP6 | NLR family, pyrin domain containing 6 | CLR11.4, NALP6, PAN3, PYPAF5 |
| NLRP9 | NLR family, pyrin domain containing 9 | CLR19.1, NALP9, NOD6, PAN12 |
| HSP90AA1 | Heat shock protein 90kDa alpha (cytosolic), class B member 1 | D6S182, FLJ26984, HSP90-BETA,  HSP90B, HSPC2, HSPCB |
| NFKB1 | Nuclear factor of kappa light polypeptide gene enhancer in B-cells 1 | DKFZp686C01211, EBP-1, KBF1, MGC54151, NF-kappa-B,  NFKB-p105, NFKB-p50, p105, p50 |
| MYD88 | Myeloid differentiation primary response gene (88) | MYD88D |
| CXCL1 | Chemokine (C-X-C motif) ligand 1   (melanoma growth stimulating activity, alpha) | FSP, GRO1, GROa, MGSA, MGSA-a, NAP-3, SCYB1 |
| CCL2 | Chemokine (C-C motif) ligand 2 | GDCF-2, HC11, HSMCR30, MCAF, MCP-1,  MCP1, MGC9434, SCYA2, SMC-CF |
| PSTPIP1 | Proline-serine-threonine phosphatase interacting protein 1 | CD2BP1, CD2BP1L, CD2BP1S,  H-PIP, PAPAS, PSTPIP |
| IKBKB | Inhibitor of kappa light polypeptide gene enhancer in B-cells, kinase beta | FLJ40509, IKK-beta, IKK2, IKKB, MGC131801, NFKBIKB |
| PYCARD | PYD and CARD domain containing | ASC, CARD5, MGC10332,  TMS, TMS-1, TMS1 |
| TXNIP | Thioredoxin interacting protein | EST01027, HHCPA78, THIF, VDUP1 |
| P2RX7 | Purinergic receptor P2X, ligand-gated ion channel, 7 | MGC20089, P2X7 |
| RAGE | Renal tumor antigen | MOK, RAGE1 |
| BIRC3 | Baculoviral IAP repeat-containing 3 | AIP1, API2, CIAP2, HAIP1, HIAP1, MALT2, MIHC, RNF49 |
| MEFV | Mediterranean fever | FMF, MEF, MGC126560,  MGC126586, TRIM20 |

**Table 2.** The list of fourteen proteins regulated by OLE from proteomics data.

| **Protein** | **Description** | **Gene Name** |
| --- | --- | --- |
| AK1 | Adenylate kinase isoenzyme 1 | AK1 |
| PEX11B | Peroxisomal membrane protein 11B | PEX11B |
| HO2 | Heme oxygenase 2 | HMOX2, HO2 |
| GNAI1 | Guanine nucleotide-binding protein G(i) subunit α-1 | GNAI1 |
| MFS10 | Major facilitator superfamily domain-containing protein 10 | MFSD10, TETRAN |
| NLRX1 | NLR family member X1 | NLRX1, NOD5, NOD9 |
| DDX51 | ATP-dependent RNA helicase DDX51 | DDX51 |
| TMA16 | Translation machinery-associated protein 16 | TMA16, C4orf43 |
| Importin-9 | Importin-9 | IPO9, IMP9, KIAA1192, RANBP9, HSPC273 |
| Calponin-2 | Calponin-2 | CNN2 |
| NOL11 | Nucleolar protein 11 | NOL11, L14 |
| SAR1α | GTP-binding protein SAR1α | SAR1A |
| SUGT1 | Protein SGT1 homolog | SUGT1 |
| TMED5 | Transmembrane emp24 domain-containing protein 5 | TMED5, CGI-100, UNQ397/PRO733 |
